# Keeping cool: A brassinosteroid receptor BRL3 helps Arabidopsis cope with heat stress

**DOI:** 10.1093/plphys/kiaf578

**Published:** 2025-11-07

**Authors:** Gunjan Sharma, Akanksha Bhatnagar

**Affiliations:** Assistant Features Editor, Plant Physiology, American Society of Plant Biologists; School of Biosciences, University of Birmingham, Edgbaston B15 2TT, UK; School of Biosciences, University of Birmingham, Edgbaston B15 2TT, UK

Climate change has increased the occurrence of frequent and intense heat waves imposing heat stress on plants and reducing crop productivity (Zhu et al. 2019; Cohen and Leach 2020). Plants naturally adapt to heat stress through thermomorphogenesis; elongating hypocotyls, roots, and petioles; and inducing early flowering and leaf hyponasty (Casal and Balasubramanian 2019). Molecular mechanisms governing thermomorphogenesis are well reported and involve the sensing of heat stress followed by signal transduction and adaptive response. Interestingly, plants also retain a memory of moderate heat exposure through epigenetic modifications, as reviewed by Staacke et al. (2025). However, the role of cell-specific sensing and signaling of heat stress is not yet fully understood.

A plasma membrane–localized leucine-rich-repeat receptor like kinase BRASSINOSTEROID-INSENSITIVE1 (BRI1) binds brassinosteroid (BR) and interacts with a co-receptor BRI1 ASSOCIATED RECEPTOR KINASE 1 (BAK1), activating downstream BR-mediated signaling. The roles of BRI1 as a growth promoter are well characterized in Arabidopsis. Interestingly, 2 other homologs of BRI namely BRI1-LIKE 1 (BRL1) and BRI1-LIKE 3 (BRL3) are predominantly expressed in vascular cells compared with BRI1, which exhibits ubiquitous expression (Cano-Delgado et al. 2004). Overexpression of BRI1 leads to drought tolerance but at the cost of a growth penalty. Surprisingly, overexpression of BRL3 confers drought tolerance without hampering plant growth (Fabregas et al. 2018). BRL3 is certainly a better candidate for engineering drought-tolerant plants because it uncouples reduced growth from drought tolerance. But how BRL3 behaves differently from its ubiquitous homolog BRI1 is still unknown.

In a recently published article in *Plant Physiology*, Rico-Medina et al. (2025) revealed that vascular localized BRL3 regulates heat stress responses. The *brl3* mutant exhibited defective thermomorphogenesis and reduced survival during heat stress exposure. Phloem-specific complementation of *brl3* mutant with wild-type (WT) BRL3 rescued the heat stress–adaptive developmental plasticity. BRL3 activates the BRI1-EMS-SUPRESSOR (BES1) transcription factor, regulating growth and development necessary for heat stress acclimation (Fig.).

**Figure.**
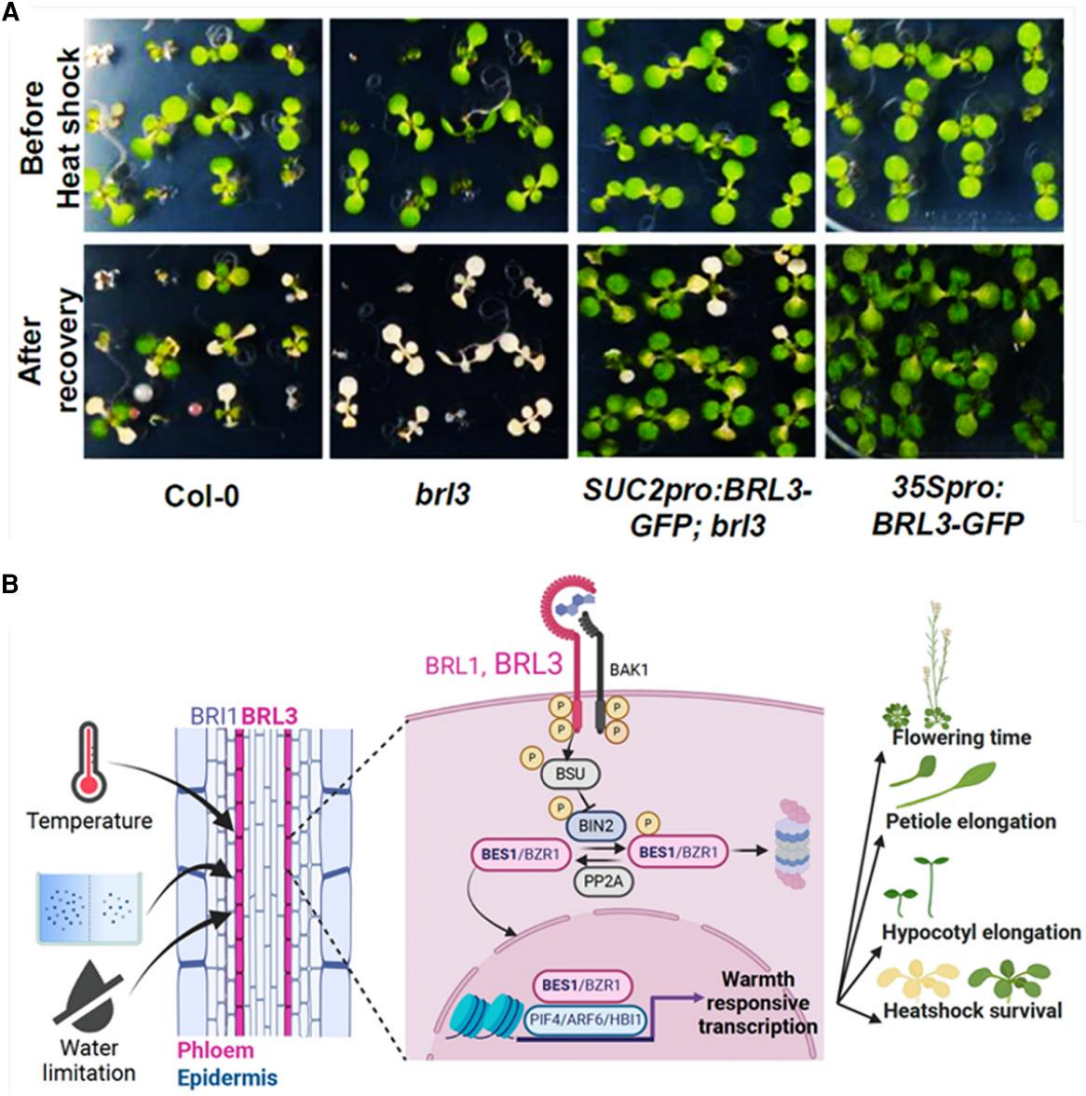
A phloem localized brassinosteroid receptor BRL3 positively regulates abiotic stress survival responses. **A)** Heat stress survival assay depicting sensitivity of *brl3* mutants to heat, while phloem-specific and constitutive expression of BRL3 rescued the phenotype. **B)** Signaling cascade of BRL3-mediated BR signaling in phloem-specific manner during heat stress. During temperature stress and water limitation, BRL3 heterodimerizes with BAK1, leading to the activation of BES1/BZR1 transcription factors and modulating the expression of warmth-responsive genes that enable plant thermomorphogenic adaptation involving flowering induction, petiole elongation, and hypocotyl elongation (adapted from Rico-Medina et al. 2025).

The authors functionally characterize the *brl3* mutant. The *brl3* mutant was phenotypically similar to WT Arabidopsis at an ambient temperature of 22 °C. However, *brl3* mutant exhibited a short hypocotyl and impaired petiole elongation when exposed to elevated temperature (28 °C) due to a reduction in cell length. This contrasts with *bri1*, which exhibits a short hypocotyl both at 22 °C and 28 °C. Interestingly, the transcript levels of BRI1 and other BRLs were unchanged in *brl3* mutant background during heat stress, indicating a distinct BRL3-mediated signaling pathway to regulate thermomorphogenic responses. The authors showed that a downstream gain-of-function *bes1* mutant rescued the growth defects of *brl3* mutant at high temperature, suggesting that BRL3 uses the same downstream pathway as BRI1. A reduced de-phosphorylation of BES1 was also observed in *brl3*. BES1 localizes to nucleus in response to BR and regulates transcription of multiple genes involved in elongation growth. A BRASSINOSTEROID-INSENSITIVE 2 (BIN2) kinase negatively regulates the BR signaling pathway by phosphorylating and destabilizing BES1 (Yin et al. 2002).

Furthermore, RNA transcriptome sequencing of *brl3* and WT shoots at 22 °C vs. at high temperature (28 °C) revealed that BRL3 targets the core thermomorphogenesis genes such as *HEAT-SHOCK TRANSCRIPTION FACTOR A7A* (*HSFA7A*), *TEMPERATURE-INDUCED LIPOCALIN* (*TIL*), and *MULTIPROTEIN BINDING FACTOR 1C* (*MBF1C*) at an elevated temperature. The *brl3* mutant was unable to induce abiotic stress-responsive genes, while over-induction of negative regulators of ethylene, carboxylic acids, and carbon-catabolic processes was observed. Carboxylic acid intermediates of the tricarboxylic acid cycle are diverted toward generating protective compounds and signaling molecules during abiotic stresses instead of catabolism-mediated energy production (Antonio et al. 2016).

The authors further investigated the spatiotemporal contribution of BRL3 in regulating elevated temperature stress responses using tissue-specific promoters in *brl3* mutant background for complementation analysis. BRL3-GFP expression under stele- (*WOLpro:BRL3–GFP*) and phloem companion cell-specific (*SUC2pro:BRL3–GFP*) promoters rescued *brl3* mutant developmental defects, while phloem-pole pericycle (*CALS8pro:BRL3–GFP*) and protophloem sieve elements (*NAC86pro:BRL3–GFP*) specific expression conferred partial rescue at 28 °C. The pericycle is a layer of cells surrounding the procambium cells, xylem, and phloem, and can be distinguished into 2 cell types based on their position relative to xylem and phloem: the xylem pole pericycle and phloem pole pericycle (Zhang et al. 2022). Interestingly, epidermis (atrichoblasts: *GL2pro:BRL3–GFP* and trichoblasts: *EXP7pro:BRL3–GFP*), endodermis (*SCRpro:BRL3–GFP*), root stem cell niche (*WOX5pro:BRL3–GFP*), and root apical meristem (*RPS5Apro:BRL3–GFP*) specific promoters failed to rescue, suggesting an inner phloem (companion cells and sieve elements) specific function. Transcriptome profiling of the *brl3* mutant complemented with phloem companion cell-specific BRL3-GFP at 22 °C vs. elevated temperature at 28 °C revealed enrichment of genes involved in photorespiration at high temperature, leading to better energy usage. Thus, BRL3 regulates growth responses to high temperature by controlling energy usage in response to hormonal stimuli.

Since BRL3 was found to modulate the abiotic stress–responsive genes in transcriptome analysis, the authors also observed the effects of heat and osmotic stresses on *brl3* mutants. The *brl3* mutant exhibited a reduced survival rate compared with the WT. However, these growth defects were restored by complementing the mutant with BRL3-GFP under the control of the phloem companion cell–specific promoter. Cumulatively, these results demonstrate the prominent role of BRL3 not only in thermomorphogenesis but also in regulating osmotic stress responses.

In conclusion, Rico-Medina et al. (2025) revealed a BRL3-mediated BR response signaling pathway specifically active in the phloem companion cells regulating cellular plasticity for temperature adaptation (Fig.). While BRI1 functions constitutively under all climate conditions, the cell type–specific expression of *BRL3* not only promotes normal plant growth but also coordinates responses to high temperature and osmotic stress. However, it is intriguing to further investigate how BRI1 and BRL3 distinctly use similar downstream signaling components. BRI1 is SUMOylated (Naranjo-Arcos et al. 2023), it would be interesting to study the post-translational modifications such as SUMOylation in regulating BRL3 protein abundance. BRL3 is a promising candidate for breeding and gene editing to produce “climate smart” crops that can survive adverse weather conditions without growth penalty.

## Recent related articles in *Plant Physiology*:

- Cackett et al. (2025) reported how the cell-specific manipulation of BR signaling can regulate the chloroplast compartment in rice bundle sheath cells.
- Zolkiewicz and Gruszka (2025) reviewed strategies for manipulating brassinosteroid homeostasis in cereals for adapting to environmental stress.

## Data Availability

No data were generated or analyzed in this study.
